# Characterization of* Clostridium difficile* Strains in British Columbia, Canada: A Shift from NAP1 Majority (2008) to Novel Strain Types (2013) in One Region

**DOI:** 10.1155/2016/8207418

**Published:** 2016-03-29

**Authors:** Agatha N. Jassem, Natalie Prystajecky, Fawziah Marra, Pamela Kibsey, Kennard Tan, Patricia Umlandt, Loretta Janz, Sylvie Champagne, Bruce Gamage, George R. Golding, Michael R. Mulvey, Bonnie Henry, Linda M. N. Hoang

**Affiliations:** ^1^British Columbia Centre for Disease Control Public Health Laboratory, Vancouver, BC, Canada V5Z 4R4; ^2^Department of Pathology and Laboratory Medicine, Faculty of Medicine, University of British Columbia, Vancouver, BC, Canada V6T 2B5; ^3^Department of Pharmacy, Faculty of Pharmaceutical Sciences, University of British Columbia, Vancouver, BC, Canada V6T 1Z3; ^4^Island Health, Victoria, BC, Canada V6T 1Z3; ^5^Providence Health Care, Vancouver, BC, Canada V6Z 1Y6; ^6^Provincial Infection Control Network of British Columbia, Vancouver, BC, Canada V6H 4B1; ^7^National Microbiology Laboratory, Winnipeg, MB, Canada R3E 3R2; ^8^School of Population and Public Health, Faculty of Medicine, University of British Columbia, Vancouver, BC, Canada V6T 1Z3

## Abstract

*Background*.* Clostridium difficile *is a major cause of gastrointestinal illness. Epidemic NAP1 strains contain toxins A and B, a deletion in repressor* tcdC*, and a binary toxin.* Objectives*. To determine the molecular epidemiology of* C. difficile *in British Columbia and compare between two time points in one region.* Methods*.* C. difficile *isolates from hospital and community laboratories (2008) and one Island Health hospital laboratory (2013) were characterized by pulsed-field gel electrophoresis, PCR-ribotyping, toxin possession,* tcdC *genotype, and antimicrobial susceptibility.* Results*. In 2008, 42.7% of isolates had NAP1 designation. Hospital-collected isolates were associated with older patients and more NAP1 types. Unlike other isolates, most NAP1 isolates possessed binary toxin and a 19 bp loss in* tcdC*. All isolates were susceptible to metronidazole and vancomycin. A 2013 follow-up revealed a 28.9% decrease in NAP1 isolates and 20.0% increase in isolates without NAP designation in one region. Then, community-associated cases were seen in younger patients, while NAP types were evenly distributed. Isolates without NAP designation did not cluster with a PFGE pattern or ribotype.* Conclusions*. Evaluation of* C. difficile *infections within British Columbia revealed demographic associations, epidemiological shifts, and characteristics of strain types. Continuous surveillance of* C. difficile *will enable detection of emerging strains.

## 1. Introduction

*Clostridium difficile* infections (CDI) are considered a top priority by Canadian and American healthcare agencies based on risk assessments of pathogens associated with antimicrobial resistance [1, 2]. CDI are characterized by diarrhea, fever, nausea, and abdominal pain and in severe cases can progress to toxic megacolon, sepsis, and death [3]. Risk factors for development of CDI include prior antimicrobial use, exposure to healthcare settings, underlying illness, and being over 65 years of age [4, 5]. In the early 2000s, outbreak-associated fluoroquinolone-resistant strains classified as B1 by restriction enzyme analysis, North American pulsotype 1 (NAP1) by pulsed-field gel electrophoresis (PFGE), and 027 by PCR-ribotyping (BI/NAP1/027) emerged and spread globally [6]. Much of our current understanding of CDI has derived from study of these strains.

*C. difficile* pathogenicity stems from the production of toxins A and B, encoded by* tcdA* and* tcdB* genes that are negatively regulated by* tcdC*, and in some isolates also binary toxin, encoded by* ctdA* and* ctdB* [3]. Mechanisms behind the enhanced virulence of BI/NAP1/027 strains remain to be fully elucidated. Hypervirulence was initially linked to increased production of toxins A and B as a result of deletions in* tcdC* but has since been challenged [7–11]. Binary toxin production is characteristic of BI/NAP1/027 strains and has been implicated in enhanced virulence but is also produced by other strains [12–16]. BI/NAP1/027 strains also are less susceptible to metronidazole, the standard antimicrobial used in CDI therapy, making treatment more complicated [17–21].

Recently, European hospitals reported changing patterns in the molecular epidemiology of CDI, suggesting that the circulation of BI/NAP1/027 strains in some areas is decreasing while other strain types are emerging [22–24]. Furthermore, the incidence of community-associated (CA) CDI is increasing across Europe and North America [25–27] and occurring in patients that are younger and healthier and have fewer of the risk factors associated with hospital-associated (HA) CDI [28–32]. The current lack of comprehensive surveillance data impedes our ability to detect changes in the molecular epidemiology of CDI [25]. Characterization of CDI in nonoutbreak settings to include CA- and HA-CDI cases would allow for a comprehensive understanding of the pathogenicity and associated markers of strains responsible for CDI and also enable the detection of early shifts in molecular epidemiology that indicate the emergence of novel outbreak strains. In the present study, we determined the molecular epidemiology of* C. difficile* infections in British Columbia during a month-long, province-wide study in 2008 that involved hospital and community specimen collection sites. A follow-up study with one of the hospital sites was carried out in 2013 to determine distribution of strain types between the two time points and between CA- and HA-CDI cases. Isolates were characterized by PFGE, PCR-ribotyping, their carriage of toxin genes and deletions in* tcdC*, and susceptibilities to antimicrobials.

## 2. Methods

### 2.1. Clinical Samples

CDI was defined by the presence of diarrhea and a positive clinical laboratory test. Hospital and community laboratories prospectively forwarded toxins A and/or B positive patient stool specimens or recovered* C. difficile* isolates to the British Columbia Public Health Laboratory for isolate confirmation, typing, and susceptibility testing. The initial study period was from March 1 to 31, 2008, and included 13 hospital laboratories, which serve mainly inpatients, and one province-wide community laboratory that serves only outpatients. These laboratories represent the majority of CDI testing within British Columbia. In 2008, all laboratories used enzyme immunoassays to detect glutamate dehydrogenase (GDH), a marker of* C. difficile*, and toxin A or B. Where confirmation was required (the clinical presentation was consistent with CDI but only the GDH result was positive or both markers of* C. difficile* were negative),* C. difficile* and toxins were detected following culture or by cell cytotoxin assay.

This study was repeated from March 3 to July 7, 2013, but only with one large hospital laboratory (LabA) that screens both hospital and community samples within the Island Health Authority for CDI by enzyme immunoassay (C. diff Quik Chek Complete; TechLab, Blacksburg, VA). This laboratory (LabA) routinely processes approximately 80% of all hospital samples and 40% of all outpatient samples originating from the region the authority covers, a region containing both rural and urban communities that represents approximately 17% of British Columbia's population. In 2013, toxin B PCR (GeneOhm Cdiff Assay; Becton, Dickinson and Co., Franklin Lakes, NJ) was used as a confirmatory test (versus cytotoxicity assay in 2008). During the 2013 study, medical record review discriminated between inpatients with CA-CDI and HA-CDI per established guidelines [27]. In the absence of other data, outpatients (*n* = 20) and inpatients characterized as having CA-CDI (*n* = 10) were collectively referred to as CA-CDI cases. Study approval was obtained from the University of British Columbia Ethics Review Board.

### 2.2. Bacterial Culture

*C. difficile* isolates were cultured on selective cycloserine cefoxitin agar containing 5% sheep blood following anaerobic incubation at 35°C for 48–72 hours. Where* C. difficile* was not initially recovered, alcohol shock treatment was performed. Typical colonies were purified on Columbia agar with 5% sheep blood (Oxoid, Nepean, ON) and* C. difficile* was identified by colony morphology, aerotolerance, and latex agglutination (*C. difficile* Latex Agglutination Kit; Microgen, Frederick, MD).

### 2.3. Molecular Typing

Pulsed-field gel electrophoresis (PFGE) was performed by digestion using 40 U* Sma*I (New England BioLabs, Ipswich, MA) based on a previously described method [33]. Bacteria within plugs were lysed at 55°C overnight with lysis buffer (content description elsewhere [33]). Plugs were then washed at room temperature 4 times at 20 minutes each, prior to digestion for 2 hours at 25°C. DNA fragments were separated using a Chef-DR III mapper system (Bio-Rad, Hercules, CA) as per established run conditions [33]. Resultant patterns were visualized on a Molecular Imager Gel Doc XR System (Bio-Rad) following gel staining with ethidium bromide and analyzed with BioNumerics software version 6.1 (Applied Maths, Austin, TX) as described previously [34] using* Xba*I digested* Salmonella braenderup* as a reference. PFGE types were assigned based on a similarity of ≥80% to NAP1-12 isolates within the Canadian Nosocomial Infection Surveillance Program* C. difficile* Database.

PCR-ribotyping was performed with high-resolution capillary gel-based electrophoresis following amplification of the 16S–23S intergenic spacer (IGS) region as previously described [35]. Briefly, amplicon fragments were identified with an Applied Biosystems 3130xl Genetic Analyzer using a capillary array in reference to GeneScan 1200 LIZ Size Standard (all Life Technologies, Grand Island, NY), and peak profiles were analyzed with BioNumerics software version 5.1 (Applied Maths, Austin, TX). Ribotypes were assigned based on profile similarity to 106 reference ribotypes within the National Microbiology Laboratory* C. difficile* database.

For toxin gene detection, DNA was extracted with InstaGene Matrix (Bio-Rad). The presence of* tcdA*,* tcdB*,* tcdC*, and* cdtB* along with the housekeeping gene* tpi* was investigated with previously described primers [9]. PCR was carried out with illustra PuReTaq Ready-To-Go PCR Beads (GE Healthcare, Piscataway, NJ) and 3 mM MgCl_2_ with an annealing temperature of 57°C on a Stratagene Robocycler (Agilent Technologies, Santa Clara, CA). Deletions in* tcdC* were detected by fragment size analysis following amplification with previously described C1 and C2 primers [36] (with C1 labeled with FAM) as described for toxin detection above. Denaturation occurred at 94°C for 5 minutes, followed by 30 cycles of 1 minute at 94°C, 1 minute at 56°C, and 1.5 minutes at 72°C. Final extension occurred for 10 minutes at 72°C. Amplicon size was determined using an Applied Biosystems 3130xl Genetic Analyzer in reference to GeneScan 1200 LIZ Size Standard. ATCC 9689 strain (locus_tag B131_00660 on NCBI GenBank accession number NZ_AQWV01000030) was used as a control.

### 2.4. Antimicrobial Susceptibility Testing

Susceptibility to clindamycin, metronidazole, and vancomycin was evaluated using E-test strips (bioMérieux, Saint-Laurent, QC) on BBL Brucella agar with 5% horse blood, hemin, and vitamin K_1_ (Becton, Dickinson and Co.) as previously described [37]. Briefly, bacteria were suspended in Brucella broth (Becton, Dickinson and Co.) prior to agar inoculation, and MICs were read after 48 hours of growth with* Bacteroides fragilis* ATCC 25285 and* Staphylococcus aureus* ATCC 29213 as controls. MIC interpretation for clindamycin and metronidazole was based on Clinical Laboratory Standards Institute (CLSI) breakpoints [38], while susceptibility to vancomycin was determined with European Committee on Antimicrobial Susceptibility Testing breakpoints [39] in the absence of established CLSI criteria.

### 2.5. Statistical Analysis

Differences between years were determined by unpaired Student's *t*-test. Differences among NAP types were determined by one-way analysis of variance (ANOVA) and Tukey's multiple comparisons test. Differences in the distribution of NAP types were determined by Pearson Chi-Square test. GraphPad QuickCalcs Software (La Jolla, CA) was used for analyses.

## 3. Results

### 3.1. 2008 British Columbia Wide* C. difficile* Molecular Epidemiology Study

In March 2008, a total of 341* C. difficile* isolates were recovered from unique CDI cases across the province of British Columbia. Of these, 271 (79.5%) and 70 (20.5%) were collected by hospital and community laboratories, respectively. Mean patient age was 69.3 years (range 0.25–99, median 77), with 230 (67.4%) being over 65 years. On average, patients presenting to community sites were 15.4 years younger, at 57.1 years, than those presenting to hospital sites (*P* < 0.0001). Data on inpatient status and case definition as either HA- or CA-CDI were not available.

NAP1 designation was identified most commonly, accounting for 146 (42.7%)* C. difficile* isolates. Other NAP types of the 12 described accounted for 122 (35.8%) isolates, while 73 (21.3%) isolates had PFGE patterns not matching a known NAP type. Isolates without NAP designation were distributed among 47 different PFGE patterns (range 1–4 isolates/pattern); the most common patterns, 0022, 0037, 0054, and 0110, were identified in only 4 (5.5%) cases each. Of the non-NAP1 NAP types, NAP2 (*n* = 35, 10.2%) and NAP4 (*n* = 46, 13.5%) designations were most common. Isolates from hospital sites had a higher proportion of NAP1 types (49.6 versus 15.7%), while isolates from community sites had a higher proportion of NAP4 (27.1 versus 9.9%) and non-NAP related types (37.1 versus 17.3%) (*P* < 0.0001). The majority of infections caused by NAP1 (*n* = 116, 79.5%) and NAP2 (*n* = 26, 74.3%) isolates occurred in patients over 65 years old, whereas other NAP types were more evenly distributed among patient age groups.

Of the toxin B gene-positive* C. difficile* isolates, 333 (97.7%) carried the gene that encodes toxin A and 170 (49.9%) carried* cdtB*, one of the two genes that encode binary toxin CDT. Further, 170 (49.9%) possessed a nonwild type genotype of* tcdC* based on gene size. Correlations between toxin gene profiles and NAP designation are shown in Table 1. Most (93.8%) NAP1 isolates possessed genes for toxin A, toxin B, and binary toxin CDT as well as a 19-bp loss in the* tcdC* gene, the latter suggestive of the described 18-bp deletion and associated single-base-pair deletion [33]. All NAP7 isolates possessed genes encoding toxins B and CDT and a 40 or 41 bp loss in* tcdC*. Of the other isolates, 105 (91%) possessed only toxins A and B genes and 55 (75.3%) had wild type size* tcdC*.

All* C. difficile* isolates were susceptible to metronidazole and vancomycin, while 31.3% were susceptible to clindamycin (Table 2). NAP1 isolates were least susceptible to metronidazole compared with isolates of other NAP types and those without NAP designation (with data from the latter combined, mean MIC 0.42 versus 0.13 *μ*g/mL) (*P* < 0.0001), while NAP2 isolates were least susceptible to clindamycin by the same analysis (mean MIC 189.2 versus 24.1 *μ*g/mL) (*P* < 0.0001).

### 3.2. *C. difficile* Molecular Epidemiology in 2008 and 2013 in One Region of British Columbia

One hospital laboratory (LabA) within the Island Health Authority was selected for follow-up in 2013. In 2008, the demographic and molecular characteristics of the 54 isolates from LabA were consistent with the overall British Columbia findings. Repeat evaluation in 2013 recovered 68* C. difficile* isolates from unique CDI cases. Patient age was similar between 2008 and 2013, with a mean/median of 71.2/79.0 years (range 1–93) in 2008 and 67.8/74.0 years (range 1–97) in 2013. In 2013, unlike in 2008, inpatient status and case definition as either HA- or CA-CDI were available for analysis. Inpatients represented 71% of cases (*n* = 48) and were significantly older than outpatients (mean 73.4 versus 54.3; *P* = 0.0017). Ten (21%) inpatients were characterized as CA-CDI cases. Overall, of the 68 inpatient and outpatient cases, 38 (55.9%) had HA infections while 30 (44.1%) had CA infections. On average, patients with CA-CDI were 16.2 years younger than those with HA-CDI, at 58.7 years (*P* = 0.0039).

NAP designation comparisons between 2008 and 2013 LabA isolates are shown in Table 3. In 2013, most isolates (*n* = 30, 44.1%) had a PFGE pattern not matching a described NAP type. The proportion of this diverse set of isolates was high in CA-CDI (50.0%) and HA-CDI (39.5%) cases, and isolates were distributed among 22 different PFGE patterns (range 1–3 isolates/pattern). The most common pattern, 0037, was identified in only 3 (10.0%) cases. Three of these non-NAP type patterns were also identified in 2008 isolates from LabA. In 2008, isolates not matching a described NAP only accounted for 24.1% of isolates (*n* = 13) and were distributed among 9 different PFGE patterns (range 1-2 isolates/pattern). Of the NAP types, NAP1 (*n* = 8, 11.8%), NAP4 (*n* = 15, 22.1%), and NAP6 (*n* = 8, 11.8%) were most commonly identified in 2013. The proportions of NAP1, NAP4, and NAP6 isolates were similar between HA-CDI (together 47.4%) and CA-CDI (together 43.3%) cases. In 2013, the majority (*n* = 14, 93.3%) of infections with NAP4 isolates occurred in patients over the age of 65, whereas only HA-CDI NAP1 isolates (*n* = 4, 50.0%) were in patients over 65 years of age. In contrast, in 2008, 22 (40.7%) isolates from LabA had NAP1 designation, while NAP4 types (*n* = 10, 18.5%) were also common. Between 2008 and 2013 there was a 28.9% decrease in NAP1 isolates and a 20.0% increase in a diverse set of isolates without NAP designation (other differences were <10%).

Other characteristics of isolates from 2013 were consistent with those from 2008 (Table 3). Most NAP1 isolates (*n* = 6, 75.0%) possessed* ctdB* for binary toxin CDT and a 19 bp smaller* tcdC*, while the NAP7 isolate did not have a detectable toxin A gene and showed a 40 or 41 bp loss in* tcdC*, and binary toxin* ctdB* was absent and* tcdC* was wild type for most isolates designated by other NAP types or without NAP designation (*n* = 49, 83.1%). All isolates were susceptible to metronidazole and vancomycin, while 22.1% were susceptible to clindamycin.

Ribotypes of 2013 LabA isolates are shown in Table 4. Most (*n* = 6, 75.0%) NAP1 isolates were designated ribotype 027, all NAP6 (*n* = 8, 100%) isolates were designated ribotype 002, and most NAP10 isolates (*n* = 3, 75%) were designated ribotype 57. NAP4 isolates were associated with various ribotypes. Generally, ribotypes were specific to NAP types; however ribotypes 014 and 153 were associated with NAP4 and NAP1 designation, respectively, as well as isolates without NAP designation. Among isolates without NAP designation, there was no dominant ribotype and no correlation between ribotype and PFGE pattern.

## 4. Discussion

Evaluation of the molecular epidemiology of* C. difficile* within British Columbia in 2008 and in 2013 revealed demographic associations, recent epidemiological shifts within one region, and characteristics of non-NAP1* C. difficile* strain types. Overall, most cases of CDI were in patients over 65 years of age (67.0%), consistent with this elderly population being at risk [4, 5]. Patients presenting to community laboratories were significantly younger than hospital patients, while patients with CA-CDI were significantly younger than those with HA-CDI. These findings align with previous reports of patients with CA-CDI being younger, likely healthier, and having fewer traditional risk factors for CDI than those with HA-CDI [28–32]. NAP1 designation was most commonly identified across British Columbia in 2008. NAP1 strains were endemic in several countries during the 2000s [6], although NAP distribution varied by region with NAP2 being predominant from 2005 to 2007 in the Canadian provinces of Manitoba [34], Ontario [40], and Prince Edward Island [41]. NAP2 and NAP4 isolates were also identified in high frequency in British Columbia in 2008. Consistent with previous reports, the presence of binary toxin, toxin A gene, and* tcdC* genotype correlated with designated NAP types [34, 40–43], although outliers did exist. NAP1 isolates were least susceptible to metronidazole [17–21].

NAP distribution shifted between 2008 and 2013 in one region in British Columbia: an increase in a diverse set of isolates without NAP designation and a decrease in NAP1 isolates were observed in the Island Health Authority region.* C. difficile* strains are naturally diverse and can evolve rapidly into those with increased pathogenicity [44, 45]. Recently, several countries have reported the emergence of non-BI/NAP1/027* C. difficile* stains within healthcare settings, including those of ribotype 078 (analogous to NAP7) [46], ribotype 244 [47, 48], ribotype 012 [49], and 018 [50]. In the present study, isolates without NAP designation were diverse and did not cluster with a particular PFGE pattern or ribotype, suggesting absence of a dominant emerging strain.

Isolates collected in 2008 from hospital sites had a higher proportion of NAP1 types compared with community site-collected isolates. While NAP1 strains were found to be more common among HA-CDI than CA-CDI in the US in 2011 [51], this difference was not seen within the one region of British Columbia that was studied here in 2013. Taken together, the high proportion of a diverse set of isolates not matching described NAP types among the 2008 community site-collected isolates and the 2013 CA-CDI isolates could relate to the transmission of strains acquired from community sources to the healthcare setting. Further analysis involving methods more discriminatory than PFGE, such as whole genome sequencing, could elucidate such a transmission pattern. Indeed, using analyses of patient interactions and whole genome sequencing, a recent study of nonoutbreak cases in a hospital setting in the UK found that distinct sources other than symptomatic CDI cases are involved in* C. difficile* transmission [52]. Therefore, the observed shift in NAP distribution between 2008 and 2013 in the Island Health Authority region could reflect the steady increase in the proportion of CA-CDI inpatient cases identified, from 11.7% in 2008 to 35.6% in 2013. The observed shifts in NAP distribution could also relate to interventions in infection control or antibiotic stewardship within healthcare settings. Indeed, such interventions did occur in the Island Health Authority between the two study periods; however their descriptions are beyond the scope of the current paper.

Our findings of population shift and strain diversity emphasize the need for continuous surveillance and characterization of circulating* C. difficile* to aid in detection and control of emerging strains. Our analysis was limited by the lack of case definition as either HA- or CA-CDI in 2008 and inclusion of only a single laboratory in 2013. Conclusions on changes in molecular epidemiology were therefore drawn from comparisons with only a subset of 2008 cases. Furthermore, we were unable to determine true cases of CA-CDI according to established criteria [27] in the absence of patient history (modified definitions were used) and CDI detection methods differed slightly between study years. Study findings are also limited by sampling periods, as CDI incidence can vary by season [53]. Comprehensive surveillance defining CA- and HA-CDI cases will enable investigations of sources of acquisition and transmission. Description of* C. difficile* isolates in nonoutbreak settings will allow for a more comprehensive understanding of the pathogenicity and associated markers of strains. Characteristics of strains that result in extensive transmission along with increased morbidity and mortality remain to be fully elucidated.

## Figures and Tables

**Table 1:** Characteristics of *C. difficile *(*n* = 341) present in British Columbia in 2008 by NAP designation (number (%)).

	NAP1 (*n* = 146)	NAP2 (*n* = 35)	NAP4 (*n* = 46)	NAP6 (*n* = 8)	NAP7 (*n* = 7)	NAP10 (*n* = 14)	Other^*∗*^ (*n* = 12)	None^†^ (*n* = 73)
Toxin gene presence								
A+ B+, CDT+	144 (98.6)	1 (2.9)	0	0	0	0	0	17 (23.3)
A+ B+, CDT−	2 (1.4)	34 (97.1)	46 (100)	8 (100)	0	14 (100)	12 (100)	55 (75.3)
A− B+, CDT+	0	0	0	0	7 (100)	0	0	1 (1.4)
*tcdC *genotype								
Wildtype	7 (4.8)	33 (94.3)	44 (95.7)	8 (100)	0	13 (92.9)	9 (75.0)	57 (78.1)
−19	137 (93.8)	2 (5.7)	0	0	0	0	0	5 (6.8)
−18	2 (1.4)	0	0	0	0	0	3 (25.0)	5 (6.8)
≥−37	0	0	0	0	7 (100)	0	0	6 (8.2)
≥+1	0	0	2 (4.3)	0	0	1 (7.1)	0	0

^*∗*^Overall distribution: 2 NAP3, 3 NAP5, 6 NAP11, and 1 NAP12. *tcdC *with an 18 bp deletion attributed to 2 NAP11 and 1 NAP12.

^†^Isolates with pulsed field gel electrophoresis patterns not matching a known NAP type.

NAP: North American pulsotype.

**Table 2:** Antimicrobial susceptibility of *C. difficile *present in British Columbia in 2008 (*µ*g/mL).

	Metronidazole	Vancomycin	Clindamycin
Susceptible number (%)	341 (100)	341 (100)	107 (31.4)
Intermediate number (%)	0 (0)	NA	89 (26.1)
Resistant number (%)	0 (0)	0 (0)	145 (42.5)

MIC range	0.016–4.0	0.125–2.0	0.25–>256
^*∗*^MIC mean	0.26	0.56	42.0
^†^MIC_90_	0.50	1.0	>256

^*∗*^Where MIC was unknown (>256 *µ*g/mL observations), 256 *µ*g/mL was used to calculate the mean.

^†^MIC required to inhibit 90% of organisms.

MIC: minimum inhibitory concentration.

**Table 3:** Comparison of *C. difficile* from LabA (number (%)).

	2008 (*n* = 54)	2013	
		HA-CDI (*n* = 38)	CA-CDI (*n* = 30)
NAP designation			
NAP1	22 (40.7)	4 (10.5)	4 (13.3)
NAP4	10 (18.5)	11 (28.9)	4 (13.3)
NAP6	2 (3.7)	3 (7.9)	5 (16.7)
Other^*∗*^	7 (13.0)	5 (13.2)	2 (6.7)
None	13 (24.1)	15 (39.5)	15 (50.0)

Toxin gene presence			
A+ B+, CDT+	24 (44.4)	6 (15.8)	9 (30.0)
A+ B+, CDT−	27 (50.0)	31 (81.6)	21 (70.0)
A− B+, CDT+	3 (5.6)	1 (2.6)	0

*tcdC *genotype			
Wildtype	27 (50.0)	29 (76.3)	21 (70.0)
−19	22 (40.7)	7 (18.4)	9 (30.0)
−18	2 (3.7)	0	0
≥−37	3 (5.6)	2 (5.3)	0

Susceptibility			
Metronidazole	54 (100)	38 (100)	30 (100)
Vancomycin	54 (100)	38 (100)	30 (100)
Clindamycin	15 (27.8)	11 (28.9)	4 (13.3)

^*∗*^2008 distribution: 2 NAP2, 3 NAP7, and 2 NAP10; 2013 distribution: 1 NAP7 (HA-CDI), 4 NAP10 (2 HA-CDI, 2 CA-CDI), and 2 NAP11 (HA-CDI).

CA-CDI: Community-associated *Clostridium difficile *infections; HA-CDI: Hospital-associated *Clostridium difficile *infections; NAP: North American pulsotype.

**Table 4:** PCR-ribotypes of *C. difficile *(*n* = 68) from LabA in 2013 by NAP designation.

NAP designation (number)	Ribotype (number)
NAP1 (8)	027 (6), 153 (1), and none^*∗*^ (1)
NAP4 (15)	011 (1), 014 (3), 020 (5), 076 (3), 354 (1), and 629 (2)
NAP6 (8)	002 (8)
NAP7 (1)	078 (1)
NAP10 (4)	057 (3), none^*∗*^ (1)
NAP11 (2)	103 (1), 106 (1)
None (27)	005 (3), 014 (4), 024 (1), 043 (1), 054 (1), 056 (4), 072 (1), 075 (3), 80 (1), 81 (1), 137 (1), 153 (1), 248 (1), 328 (1), 404 (1), and none^*∗*^ (5)

^*∗*^Isolates with 16S–23S intergenic spacer region profiles not matching a known ribotype.

NAP: North American pulsotype.
